# Management of mountainous meadows associated with biodiversity attributes, perceived health benefits and cultural ecosystem services

**DOI:** 10.1038/s41598-019-51571-5

**Published:** 2019-10-18

**Authors:** Raja Imran Hussain, Ronnie Walcher, Renate Eder, Brigitte Allex, Peter Wallner, Hans-Peter Hutter, Nicole Bauer, Arne Arnberger, Johann G. Zaller, Thomas Frank

**Affiliations:** 1 Institute of Zoology, Department of Integrative Biology and Biodiversity Research, University of Natural Resources and Life Sciences, Vienna, Austria; 2 Institute of Landscape Development, Recreation and Conservation Planning, Department of Spatial, Landscape and Infrastructural Sciences, University of Natural Resources and Life Sciences, Vienna, Austria; 3 Department of Environmental Health, Center for Public Health, Medical University, Vienna, Austria; 4 Swiss Federal Institute for Forest, Snow and Landscape Research (WSL), Economics and Social Sciences, Social Sciences in Landscape Research, Zurich, Switzerland

**Keywords:** Ecosystem services, Grassland ecology

## Abstract

Associations between biodiversity, human health and well-being have never been discussed with reference to agriculturally managed, species-rich mountainous meadows. We evaluated these associations between extensively managed (one mowing a year, no fertilization) and abandoned (no mowing since more than 80 years, no fertilization) semi-dry meadows located in the Austrian and Swiss Alps. We quantified the richness and abundance of plants, grasshoppers, true bugs, bumblebees, syrphids and landscape characteristics in the surroundings of the meadows. Associations between these biodiversity attributes and short-term psychological and physiological human health effects were assessed with 22 participants (10 males, 12 females; mean age 27 years). Participants´ pulse rate, systolic blood pressure (SBP) and diastolic blood pressure (DBP) were not affected during visits to managed or abandoned meadows. However, perceived health benefits (e.g., stress reduction, attention restoration) were higher during their stays in managed than in abandoned meadows. Also, the attractiveness of the surrounding landscape and the recreation suitability were rated higher when visiting managed meadows. Perceived naturalness was positively correlated with plant richness and flower cover. A positive correlation was found between SBP and forest cover, but SBP was negatively correlated with the open landscape. A negative association was found between grasshoppers and recreational and landscape perceptions. We suggest to discuss biodiversity attributes not only in connection with agricultural management but also with cultural ecosystem services and health benefits to raise more awareness for multifaceted interrelationships between ecosystems and humans.

## Introduction

The association between biodiversity, ecosystems services and human health and well-being has gained increased consideration in global scientific and political debates in the past few years^1–3^. European alpine grasslands have been extensively managed for hundreds of years by local farmers and traditional land-use, bearing high plant and insect diversity^4,5^. However, changes in agricultural practices and low farm incomes have resulted in abandonment of these alpine grasslands^6–9^. Mountainous meadows do provide restorative benefits^10^ and are considered to promote human health^11^. However, there is a lack of direct evidence in linking biodiversity, ecosystem services, human health and well-being with meadow management intensity.

Ecosystems offer services valuable to human health and well-being positively affecting mental health, the cardiovascular system and stress levels^12–18^. Although biodiversity plays a key role for delivering ecosystem and regulating services^19^ and is important for human health and well-being^20^, relationships between biodiversity and human health and well-being are not uniform^21,22^. Fuller *et al*.^23^ assumed that plants are the most evident and stationary element of biodiversity. They are visible to people and may be useful indicators for the relationships between biodiversity and human health^24^. Several studies found positive associations between perceived health benefits and birds or plant diversity^25,26^ while others did not find a consistent relationship between plant richness and psychological benefits^27^. Further investigations are required to clarify potential knowledge gaps on the association between biodiversity and human health^21,28^.

Studies on the associations between the biodiversity, physiological and well-being benefits and the degree of naturalness and human health were primarily carried out in the urbanized context or only on specific organisms such as butterflies^29^ or birds^3^. Our explorative study for the first time aims in getting an understanding on the relationships between biodiversity and human health on meadows by considering several insect groups. While butterflies are very obvious to humans and well investigated^23,25^, other pollinators like bumblebees, true bugs, grasshoppers and syrphids have not been investigated so far in that context, although these insects are characteristic elements of the fauna of alpine meadows. Grasshoppers and true bugs, for example, are abundant species in meadows and can be experienced by the people in many ways, namely at tactile, visual and audible perception levels. When people sit in the meadow, they can easily interact with insect groups.

The objective of the present work was to collectively evaluate associations between several biodiversity attributes, landscape characteristics and cultural ecosystem services on human health and well-being and how much agricultural management intensity affects these associations. The goal of our study was to explore the association between health and well-being benefits and measured biodiversity attributes in two grassland management regimes. In contrast to previous studies^23,30^, our study relied on both perceived and measured health benefits when correlating these with several biodiversity attributes of alpine meadows. We hypothesized that meadows with a higher biodiversity would reduce stress levels and other psychological health parameters that would further translate into measurable physiological health benefits. The investigations were carried out with 22 participants (10 males, 12 females; mean age 27 years) in mountain landscapes in the Austrian and Swiss Alps. Biodiversity attributes and landscape parameters were assessed by standardized sampling methods.

## Results

### Species and plant richness

In total, we found 12 grasshopper, 16 true bug, 8 syrphid, 5 bumblebee and 115 plant species in both managed and abandoned meadows (Supplementary Table S1). *Bromus erectus* was the dominant plant species in managed meadows. The grasses *Brachypodium pinnatum*, *Molinia caerulea* and the non-leguminous herb *Laserpitium latifolium* were the dominant species in abandoned meadows.

**Figure 1:**
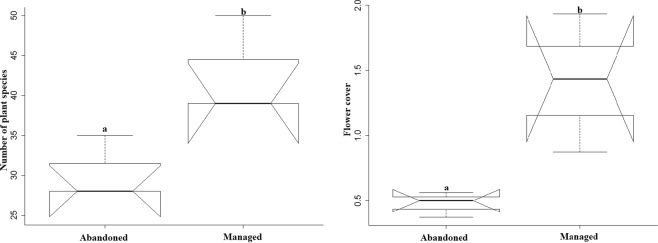
Plant richness and flower cover in abandoned and managed meadows. Dissimilar letters above box plots show significant differences.

Plant richness (F_1,6_ = 8.053, p = 0.047) and flower cover (F_1,6_ = 9.04, p = 0.027) differed significantly between managed and abandoned meadows (Fig. 1). Species richness and abundance of true bugs, grasshoppers, syrphids and bumblebees were non-significant between management types, as was also for surrounding landscape (Supplementary Table S2). However, there was a slight trend of increasing grasshopper abundance in managed meadows. When data of three study sites were pooled together then we found a significant difference only in bumblebees’ abundance and richness between Austria and Switzerland study sites (Supplementary Fig. S1).

### Cardiovascular parameters and perceptions of participants

We tested whether there are differences in pulse rates and blood pressure (SBP, DBP), perceived health benefits, recreation and landscape related attributes between managed and abandoned meadows. Overall, participants perceived various health benefits (stress reduction, attention restoration, increase in well-being) during their stay in the meadows (Table 1). We run a repeated measures analysis (including site, order of visits and management type) and found that the order of visits (main effect, as well as interaction effects) had no influence on the perceptions of naturalness (p > 0.05).

**Table 1:** Perceived health effects and cultural ecosystem services in two management types across Austrian and Swiss Alps.

Perceived health benefits and ecosystem services (Mean)	Abandoned meadow	Managed meadow
Stress reduction*	2.03	1.80
Attention restoration*	2.13	1.99
Change in well-being*	1.86	1.70
Landscape beauty*	2.25	1.43
Naturalness*	2.36	1.21
Suitability for recreation*	2.52	2.25
Noise perception-site*	2.89	3.07
Noise perception-background*	2.67	2.82
Probability to revisit	2.68	2.33
Meadow beauty	2.25	1.43

Answer scales: Stress reduction (1 = very good, 5 = absolutely not); attention restoration (1 = very good, 5 = absolutely not); change in well-being (1 = enhanced, 3 = unaffected, 5 = decreased); landscape beauty and naturalness (1 = very good to 5 = absolutely not); suitability for recreation (1 = very appropriate, 5 = not appropriate); noise perception-site (1 = noiseless to 5 = very intense); noise perception-background (1 = very pleasing to 5 = not pleasant); probability to revisit and meadow beauty (1 = very good to 5 = absolutely not).

*Significant at the 0.05 probability level.

The results of the GLMs with repeated measures showed that there were no significant differences between the managed and abandoned meadows in pulse rate (F_1,22_ = 0.655, *p* = 0.300), SBP (F_1,22_ = 0.599, p = 0.448) and DBP (F_1,22_ = 0.064, *p* = 0.803). Significant differences were found for perceived stress reduction, attention restoration, well-being and degree of naturalness between managed and abandoned meadows (stress reduction F_1,22_ = 15.464, *p* = 0.001; attention restoration F_1,22_ = 5.047, *p* = 0.036; well-being (F_1,22_ = 6.687, *p* = 0.017), degree of naturalness (F_1,22_ = 36.713, *p* < 0.001), suitability for recreational purposes (F _1,22_ = 4.309, *p* = 0.050), landscape beauty (F_1,22_ = 7.117, *p* = 0.014), and noise perceptions (F_1,22_ = 5.318, *p* = 0.031). No differences were found for beauty of the meadow and the probability of a revisit.

### Relationships between biodiversity and cardiovascular health and landscape perceptions

**Figure 2:**
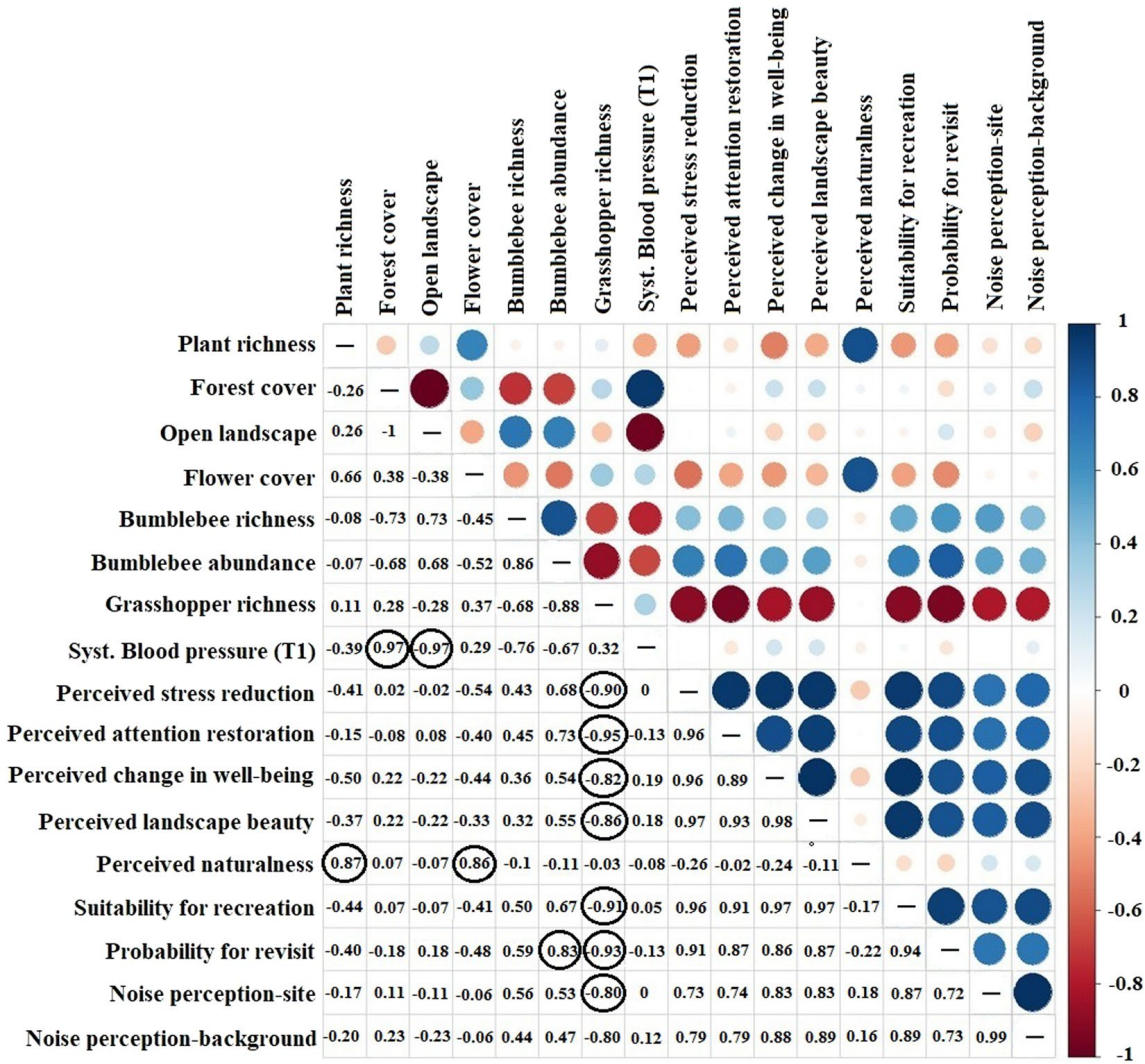
Correlation matrix showing co-occurrences of biodiversity attributes, landscape characteristics, cultural ecosystem services and human health and well-being. Negative correlations in red color and positive correlations are presented in blue. The size of the circle and color intensity is relative to the correlation coefficients. In the right side of the matrix, the legend color shows the corresponding colors and the correlation coefficients. Statistically significant correlations are marked by circles.

Perceived naturalness was positively correlated with number of plant species (r = 0.87, *p* = 0.023) and flower cover (r = 0.86, *p* = 0.028). We also detected a positive correlation between systolic BP (T1) and forest cover within the 500 m radius (r = 0.973, *p* = 0.001), and between the probability to revisit a meadow and bumblebee abundance (r = 0.82, *p* = 0.043). Systolic BP (T1) was negatively correlated with level of openness of the landscape (r = −0.969, *p* = 0.001). Grasshopper richness was negatively correlated with perceived stress reduction (r = −0.89, *p* = 0.01), attention restoration (r = −0.09, *p* = 0.004), change in well-being (r = −0.82, *p* = 0.043), landscape beauty (r = −0.85, *p* = 0.028), suitability for recreation (r = −0.90, *p* = 0.012), noise perceptions (r = −0.80, *p* = 0.05) and probability to revisit meadows (r = −0.93, *p* = 0.006) (Fig. 2). No correlations were found between human health perceptions and vegetation cover, true bugs and syrphids diversity (p > 0.05).

## Discussion

To the best of our knowledge, this is the first study which analyzed the relationships between measured biodiversity attributes and perceived as well as measured human health benefits among alpine grassland management regimes. Extensively managed alpine grasslands are biodiversity hotspots of mountain landscapes, however are steadily disappearing due to abandonment. We found several negative and positive correlations between biodiversity attributes and human health and well-being and cultural ecosystem services. Agricultural management appeared to only play a minor role in this context.

Managed and abandoned meadows are characterized by different plant richness. Our results highlight that perceived naturalness has an association with plant richness. In our study, we followed Fuller *et al*.^23^ who stated that plants are the most evident and stationary element of biodiversity. When management has an influence on plant richness and plant richness affects perceived naturalness, then there is actually also an indirect effect of management. Previous research has shown that people have a positive response to higher plant richness^31^, although recognizing plant richness just by sighting itself can be quite imprecise^32^, while Qiu *et al*.^33^ found that lay people can recognize differences in biodiversity within an urban green space. Perceived naturalness was positively related to plant richness and flower cover, which suggests that when participants are in managed meadows, that contain high plant richness and flower cover, they perceive more naturalness^29^.

Flowers have been an indicator as social, ravishing, spiritual and emotional symbol^34^. People often benefit from contacts with images of flowers, as well as advantages from interaction with real nature^35^. Higher flower cover in managed meadows seems representative of perceived naturalness by the participants not only due to their influential effect on mood^36^ but also on health and well-being^37^. However, in contrast to previous studies^38,39^, plant richness and flower cover were not related to human health. This might be due to healthy participants with similar age and same cultural background used in the study design.

Several studies revealed a decline in blood pressure in natural or semi-natural grasslands linked to urban settings^40,41^, while others were unable to find such patterns^42,43^. In the present study we did not find differences between management regimes but found a negative relationship between SBP and openness of the landscape and a positive relationship between surrounding forest cover and SBP, indicating that in a more open landscape the SBP is lower.

All managed meadows provided higher self-reported health benefits (stress reduction, attention restoration, change in well-being), naturalness and cultural ecosystem services (landscape beauty, suitability for recreation and noise perception) assuming that a stay in managed meadows is more beneficial for mental and physiological restoration than a stay in an abandoned meadow. Although participants valued positively both management regimes in terms of beauty and probability to revisit, managed meadows, richer in flower cover and plant species than abandoned meadows, were rated higher. This also showed that mountainous meadows differ in their perceived health effects^10^.

A consistent negative relationship was found between grasshopper diversity and many landscape quality and health perceptions. Andujar^44^ observed that grasshopper noises were not pleasant to those people who are uninspired by insects. Besides producing sounds, adult grasshoppers fly and jump, and combined with their high numbers, become more noticeable to participants compared to most other species living in the meadows. The occurrence of different grasshoppers flying around and on participants while they were sitting in the meadows may have caused a disordered impression. We think this might have evoked some biophobia^45^ of grasshoppers to participants^46^. Previous research has also shown that landscapes evaluated as chaotic, confusing and very complex are not perceived as restorative environments, which may not provide health benefits to humans^47^. This might explain why participants discouraged the presence of grasshoppers for stress reduction, attention restoration, landscape beauty and suitability for recreation. Instead, participants preferred to revisit the meadows in the presence of many bumblebees. We assume that for our participants a good quality of life emerges from the functioning of pollinators as sign of identity and an aesthetically significant factor in landscapes^48^. We also assume that participants’ aesthetic appreciation and naturalness were linked with the visual observation of biodiversity attributes^49^. The interactions of grasshoppers and bumblebees with participants made participants more perceptive, compared to the less visible syrphids and true bugs.

A stay in these meadows positively influenced the subjective well-being of participants, confirming previous research on restorative effects of natural and semi-natural areas on human health and well-being^14–18^. However, participants perceived higher health benefits during their stay in managed meadows than abandoned meadows. The beauty of managed meadows and the surrounding landscape were rated higher, although the surrounding landscape was the same for each meadow pair. One explanation might be that managed meadows were richer in flower cover. Flower cover may be the most noticeable visual cue between the two managements through which participants perceived variations in the environment around them^23^, and may be the main factor influencing their perceptions of biodiversity. Another explanation for the higher health benefits of managed meadows might be that participants had perceived lower noise levels in abandoned meadows.

**Figure 3:**
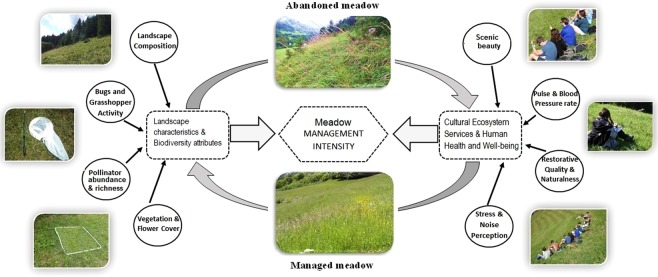
Conceptual framework illustrating links between the impacts of biodiversity attributes, landscape characteristics, cultural ecosystem services and human health and well-being. These services are interconnected by influential impact of two mountain management regimes.

Biodiversity attributes revealed some relationships with cultural ecosystem services and perceived health benefits. However, these relationships might get obscured in the absence of the two management types (Fig. 3). Managed meadows, rich in plant species and flowers, provide more cultural ecosystem services but not more measured health benefits compared to abandoned meadows. Further expansion of our study by increasing participant numbers and having greater variety of cultural and educational backgrounds would also help to reveal additional aspects that might alter associations between biodiversity attributes and perceived and measured health benefits in areas with different agricultural management intensities.

## Conclusion

We conclude that several biodiversity attributes were associated with cultural ecosystem services and health benefits within two studied grassland management regimes. These relational effects have been addressed the first time by means of visiting managed and abandoned meadows of Austrian and Swiss Alps. Although humans probably cannot directly sense ecological quality^50^, there is a trend built on perceptions, that ecological quality has some connections with grassland management. Managed meadows having high plant richness and flower numbers increased the perception of naturalness which highlights their importance as natural element. Our conclusion not only helps to promote grassland management but also indicates its significant cultural value for human health and well-being. We suggest to discuss biodiversity attributes not only in connection with agricultural management intensity but also with cultural ecosystem services and health benefits in order to raise more awareness for the importance of interrelationships between ecosystems and humans.

## Material and Methods

### Study regions

The research was carried out in August 2015 in three UNESCO-LTSER biodiverse regions of the Austrian and Swiss Alps (Table 2). The study sites were six semidry meadows, three of them managed and three abandoned, comprised in the regions Eisenwurzen (M1: Styria, Austria), the Großes Walsertal Biosphere Reserve (M2: Vorarlberg, Austria) and the Val Müstair Biosphere Reserve (M3: Graubünden, Switzerland). Managed meadows were mown annually since more than 80 years, usually between mid-July and the beginning of August. Abandoned sites were between 20 and 40 years old. The sizes of abandoned meadows ranged between 300 and 4300 m^2^, the sizes of managed meadows ranged between 470 and 5150 m^2^. We did not find any significant differences between sizes of meadows for each of the three regions (ANOVA: M1: F = 1.832, *p* = 0.25; M2: F = 3.561, *p* = 0.13; M3: F = 1.378, *p* = 0.31). In all three regions, the managed and the abandoned meadows were bordering, separated from each by some trees or tree lines.

**Table 2:** Study sites and their regional characteristics along an altitudinal gradient in Austria and Switzerland.

Country/Federal State	Region	Municipality	Mean temperature in August	Mean annual rainfall	Altitude a.s.l*	GPS coordinate
Austria/Styria	Eisenwurzen	Pürgg	20 °C	1088 mm	790 m	47°31′N, 14°03′E
Austria/Vorarlberg	Großes Walsertal	Sonntag/Buchboden	23 °C	1633 mm	1200 m	47°14′N, 09°57′E
Switzerland/Graubünden	Val Müstair	Tchierv	25 °C	811 mm	1740 m	46°36′N, 10°20′E

*Above sea level.

### Biodiversity attributes measurement

Syrphid species richness and abundance were surveyed using two different methods: line transects and observation plots^51,52^. For the line transect method, three transects were established in each meadow. Each transect was 15 m long and 2 m wide and the distance between each transect was 10 m. Additionally in each meadow, four 2 m^2^ observation plots were selected in a straight line at distances of 0 m, 3 m, 9 m and 27 m. Observations were recorded over a period of 15 minutes for each plot. The two methods were carried out simultaneously in each meadow, therefore, field conditions during sampling were similar.

The data obtained from both methods were combined per study site. Since the survey was carried out on six meadows (three managed and three abandoned meadows), the number of statistical units was 6 in the analysis. We sampled bumblebees on four 20 m^2^ study plots in each meadow for 15 minutes. Every individual bumblebee specimen was collected by sweep netting and counted. Bumblebees were set free on-site after identification^8^. Heteropteran bugs were sampled by sweep netting. We applied a total of 90 sweeps separated in 3 × 30 sweeps in the center of each meadow^53^. Species identification was performed in the laboratory using a taxonomic key provided by Wagner^54^ and Strauss^55^. Grasshoppers were assessed with recording devices, which considered as an appropriate method for recording grasshoppers richness within their habitat^56^. We attached a bat detector (heterodyne) to the recording device to enhance sounds (Batbox III D). Later, grasshoppers were identified at species level by listening using auditory assessment material from the field^57^.

### Plant and landscape parameters

Vegetation and flower cover were based on the estimation of how much area a plant or flower covers in a defined area. This method also assessed the amount of canopy cover that occurred on a study site^58^. Plant species richness, vegetation cover (%) and flower cover (%) were assessed by four 1 m^2^ (1 × 1 m) frames in each meadow. Each frame was subdivided into 4 sections of 0.25 m^2^ and the distance between each frame was 5 m. The frame was set on the highest level of the vegetation and every plant species within each section was identified^52,59^. Later, plant community data per study site were used in the analysis. At each of the managed and abandoned sites an area was selected which was fairly uniform with respect to topography, soil and vegetation. This procedure was adopted randomly in June and August on each study site to measure vegetation representative of each meadow. This also ensured that late-flowering species had not been missed. Vegetation cover and flower cover were estimated using a modified Braun- Blanquet scale^60^ for species cover^61,62^.

Surrounding landscape structure (forest cover and open landscape) was assessed within a 500 m radius around the center of each study site by means of GIS^63^ (geographical information system). In every circle, the percentage of open landscape and forest cover were calculated in ArcGIS (basemap). Open landscape was almost entirely covered by grassland. All sampling was conducted between 10 a.m. to 5 p.m. when climatic conditions were suitable, i.e. minimum temperature 15 °C, no rain or wind and dry vegetation.

### Human health and recreational effects measurements

According to the World Health Organisation of the United Nations (WHO) health is a state of physical, mental and social well-being and not merely the absence of disease or infirmity; well-being is described as the state of being relaxed and healthy^19,64^. A sample of 22 healthy participants, balanced in gender (10 males, 12 females) and of a fairly similar age (mean age = 27, ranging from 22 to 36 years; non-smokers), was used for the assessment of short term effects on human well-being and recreation. The study fulfilled national ethical requirements and participants gave informed written consent before they joined the study. Also, the study was performed in accordance with the Declaration of Helsinki, and the study protocol was approved by the ethics committee of the Earth System Sciences (ESS) programme. Participants were briefed about the objective of the study, methodology and associated issues. The participants consisted of employed individuals and students from different Austrian institutions with similar culture background and educational levels. For the chosen research design and sample size, we expected that the test strength would be adequate^65^.

The participants visited in different sessions each meadow in a standardized manner^17,18^ at very similar weather conditions. Two separate visits per meadow were made in order to obtain more robust results. Participants started with the abandoned meadow in the morning of the first day, and visited the managed one in the afternoon. Next day, we reversed the visit order, i.e. the managed meadow was visited in the morning and the abandoned one in the afternoon. At the next region, we started with the abandoned meadow in the morning, followed by the managed one in the afternoon.

The approximate duration of a single visit was about two and a half hours at each study site. Participants’ blood pressure (t1) and pulse rates were measured on the study site, after a bus ride of between 25–30 minutes. On reaching at the meadow, after a very easy 10-minute walk or shuttle transport, participants then sat and perceived the study sites for 15 minutes, after that we note down blood pressure (t2) and pulse rates. Then participants perceived the landscape over again for few minutes and completed several questionnaire forms. When participants were sitting in the meadows they were directly exposed to the environmental conditions perceiving plants and insects with all their senses in short distances. The difference between pulse and blood pressure were indicated as T1 (t1 − t2). The same procedure was followed at all study sites.

To test physiological factors of the cardiovascular system (pulse, systolic (SBP) and diastolic blood pressure (DBP)), self-inflating blood pressure cuffs (boso medilife S) were used. Over the years, research has found that both SBP and DBP are equally important in monitoring heart health. Greater risk of heart disease related to higher systolic pressures (>130 mm Hg)^66^.

Pulse, SBP and DBP were recorded when sitting in upright position, quietly for 5 minutes prior to measurement. Talking was not allowed. We measured pulse, SBP and DBP three times per measurement at each study site. We used the third (most reliable) measurement of pulse and blood pressure for analyses^22^. During each visit, noise levels were permanently checked using measurement device (Voltcraft SL-451). Based on 30 minutes of observations, an average noise level was recorded for each study site.

### Questionnaire

Perceived health benefits (i.e. stress relief, well-being, and attention restoration) were evaluated using 5-point response scales. Participants were questioned whether a visit in the meadow had reestablished their attention (1 = very good, 5 = absolutely not), decreased their stress (1 = very good, 5 = absolutely not), and altered their psychological well-being (1 = enhanced, 3 = unaffected, 5 = decreased). Landscape quality indicators included perceptions of naturalness and sound, and the attractiveness of the neighboring landscape and of the meadows applying 5-point response scales. Landscape and study site attractiveness were judged by a response scale ranged from 1 = very good to 5 = absolutely not. The answer scale of the sound level perception ranged from 1 = noiseless to 5 = very intense; background sound ranged from 1 = very pleasing to 5 = not pleasant. Participants had to estimate the appropriateness of study site for recreation (1 = very appropriate, 5 = not appropriate), and if they would visit again this study site on a scale from 1 = very good to 5 = absolutely not.

### Statistical analysis

In the first step, all biodiversity attributes and physiological human health data were analyzed for normal distribution and homogeneity of variance to fulfill the prerequisite for ANOVA by using the Levene’s test and box plots. We used General Linear Models (GLM) to analyse differences in perceived health effects, blood pressures and pulse rates between the study sites with Poisson error distribution (corrected by quasi-poisson when there was overdispersion). Pearson rank correlation was computed to assess the relationship between different biodiversity attributes (species numbers of true bugs, syrphids, plants, grasshoppers, and bumblebees), landscape characteristics (percentage of open land, forest and vegetation cover) and perceptions of cultural ecosystem services (naturalness, recreation, landscape beauty, noise perception) as well as perceived health benefits, blood pressure and pulse. Previous research analyzing relationships between biodiversity and human health and well-being typically relied on correlations^3,25,26^. The *corrplot* package was used for graphical presentation of correlation coefficient matrix because it contains algorithms to do matrix reordering. All statistical calculations were performed using R version 3.3.1^67,68^ by using an alpha level of 0.05.

## Supplementary information

Supplementary material

## Data Availability

All data generated or analyzed during this study are included in this published article (and its Supplementary Information Files).
