# Supplementary material for: Management of mountainous meadows associated with biodiversity attributes, perceived health benefits and cultural ecosystem services
Source: Sci Rep. 2019 Oct 18;9:14977. doi: 10.1038/s41598-019-51571-5 (PMC6802121; doi:10.1038/s41598-019-51571-5)
Supplement: Supplementary file 1 — Supplementary material [file 41598_2019_51571_MOESM1_ESM.pdf]

Management of mountainous meadows associated with biodiversity attributes, perceived health benefits and cultural ecosystem services

Raja Imran Hussain[1*], Ronnie Walcher[1], Renate Eder[2], Brigitte Allex[2], Peter Wallner[3], Hans-Peter Hutter[3], Nicole Bauer[4], Arne Arnberger[2], Johann G. Zaller[1], Thomas Frank[1]

[1]Institute of Zoology, Department of Integrative Biology and Biodiversity Research, University of Natural Resources and Life Sciences Vienna, Austria

[2]Institute of Landscape Development, Recreation and Conservation Planning, Department of Spatial, Landscape and Infrastructural Sciences, University of Natural Resources and Life Sciences Vienna, Austria

[3]Department of Environmental Health, Center for Public Health, Medical University Vienna, Austria

[4]Swiss Federal Institute for Forest, Snow and Landscape Research (WSL), Economics and Social Sciences, Social Sciences in Landscape Research, Switzerland

* Corresponding author
Present address: Institute of Zoology, Department of Integrative Biology and Biodiversity Research, University of Natural Resources and Life Sciences, Vienna, Austria
Gregor-Mendel-Straße 33/I
1180 Wien
e-mail: raja.hussain@boku.ac.at
Phone : +4368860251279

Supplementary Material:

Supplementary Table S1: List of species of biodiversity attributes in two management types across Austrian and Swiss Alps. Species presence is marked with "X" while empty space indicates absence.

Biodiversity attributes	Species list	Abandoned Meadow	Managed Meadow
Grasshoppers	*Chorthippus biguttulus*	X	X
	Decticus verrucivorus		X
	Euthystira brachyptera	X	X
	Gomphocerippus rufus	X	
	Metrioptera brachyptera	X	X
	Omocestus rufipes		X
	Pholidoptera griseoaptera	X	X
	Chorthippus parallelus	X	X
	Psophus stridulus	X	X
	Roeseliana roeselii	X	X
	Stenobothrus lineatus		X
	Tettigonia cantans	X	
True Bugs			
	Carpocoris melanocerus	X	
	Carpocoris purpureipennis	X	X
	Cymus glandicolor	X	
	Graphosoma lineatum	X	X
	Megalonotus chiragra		X
	Myrmus miriformis	X	
	Nabis ferus	X	
	Orthops kalmii	X	X
	Palomena prasina	X	X
	Rhopalus subrufus	X	
	Spilostethus saxatilis	X	X
	Stenodema holsata	X	
	Stenodema laevigata	X	
	Stenodema sericans	X	
	Stictopleurus crassicornis		X
	Stictopleurus punctatonervosus		X
Syrphids			
	Cheilosia impressa	X	
	Epistrophe diaphana		X
	Episyrphus balteatus	X	X
	Eupeodes lapponicus	X	X
	Melanostoma mellinum	X	X
	Pipizella varipes		X
	Pipizella virens		X
	Syritta pipiens	X	X

Bumblebees			
	Bombus humilis	X	
	Bombus lapidarius	X	X
	Bombus pascuorum	X	
	Bombus mucidus		X
	Bombus pratorum		X
Vascular Plants			
	Achillea millefolium agg.	X	X
	Aegopodium podagraria	X	
	Ajuga genevensis		X
	Alchemilla monticola	X	X
	Allium carinatum	X	X
	Allium lusitanicum		X
	Anthericum liliago	X	
	Anthoxanthum odoratum	X	X
	Anthriscus sylvestris		X
	Anthyllis vulneraria carpatica		X
	Aquilegia atrata	X	
	Arabis hirsuta		X
	Arenaria serpyllifolia		X
	Arrhenatherum elatius	X	X
	Avenula pubescens pubescens	X	X
	Brachypodium pinnatum	X	X
	Briza media	X	X
	Bromus erectus	X	X
	Buphthalmum salicifolium	X	X
	Campanula rapunculoides	X	
	Campanula rotundifolia	X	
	Carduus defloratus viridis		X
	Carex alba	X	
	Carex ericetorum	X	X
	Carex flacca		X
	Carex spicata	X	X
	Carex sylvatica		X
	Carlina acaulis acaulis	X	X
	Centaurea jacea	X	X
	Centaurea scabiosa scabiosa	X	X
	Cerastium holosteoides		X
	Chaerophyllum aureum	X	
	Clinopodium vulgare	X	X
	Colchicum autumnale	X	X
	Convolvulus arvensis	X	

Cruciata laevipes	X	
Cynosurus cristatus		X
Dactylis glomerata	X	X
Dianthus carthusianorum		X
Dianthus deltoides	X	
Epipactis atrorubens	X	
Euphorbia cyparissias		X
Festuca pratensis		X
Festuca rubra rubra	X	X
Festuca rupicola	X	X
Festuca valesiaca	X	X
Fragaria viridis	X	X
Fraxinus excelsior	X	X
Galium album	X	X
Galium pumilum	X	X
Gentianella aspera		X
Geranium pratense	X	
Glechoma hederacea		X
Gymnadenia conopsea		X
Helianthemum nummularium obscurum	X	X
Hepatica nobilis	X	
Heracleum sphondylium		X
Hieracium pilosella		X
Hieracium spec.	X	X
Hippocrepis comosa	X	X
Hypericum perforatum	X	
Knautia arvensis arvensis		X
Knautia drymeia intermedia		X
Koeleria pyramidata pyramidata		X
Laserpitium krapfii gaudinii	X	
Lathyrus pratensis	X	X
Leontodon hispidus hispidus		X
Leucanthemum ircutianum		X
Linum catharticum		X
Lotus corniculatus	X	X
Medicago falcata	X	X
Melampyrum sylvaticum	X	
Molinia caerulea	X	
Onobrychis montana		X
Phyteuma orbiculare	X	X
Pimpinella saxifraga saxifraga	X	X
Plantago lanceolata		X
Plantago media		X

Poa angustifolia	X	X
Poa trivialis		X
Polygala comosa		X
Polygonatum odoratum		X
Potentilla erecta	X	X
Primula elatior	X	X
Prunella grandiflora	X	X
Ranunculus bulbosus		X
Ranunculus nemorosus		X
Rhinanthus alectorolophus alectorolophus		X
Rhinanthus minor		X
Rosa spec.	X	
Rumex acetosa	X	X
Salvia verticillata	X	X
Sanguisorba minor	X	X
Scabiosa columbaria		X
Scabiosa lucida		X
Silene nutans nutans		X
Silene vulgaris vulgaris		X
Sorbus aucuparia	X	
Taraxacum officinale agg.		X
Teucrium chamaedrys	X	
Thymus pulegioides pulegioides	X	X
Tragopogon pratensis		X
Trifolium dubium		X
Trifolium medium medium	X	
Trifolium montanum		X
Trifolium pratense pratense		X
Trifolium repens		X
Trisetum flavescens	X	X
Vaccinium myrtillus	X	
Valeriana officinalis	X	X
Veronica chamaedrys chamaedrys	X	X
Vicia cracca	X	X
Viola hirta	X	
Viola rupestris	X	
Viola spec.	X	

Supplementary Table S2: Number of statistical units (N), degrees of freedom (df), F and P-values showing the effects of management on true bugs, grasshoppers, syrphids and bumblebees.

		Management			
		N	df	F	P
**True bugs**					
	Total species	6	4	0.73	0.44
	Total individuals	6	4	1.88	0.24
**Grasshoppers**					
	Total species	6	4	0.10	0.77
	Total individuals	6	4	0.18	0.76
**Syrphids**					
	Total species	6	4	0.21	0.67
	Total individuals	6	4	0.18	0.80
**Bumblebees**					
	Total species	6	4	0.10	0.90
	Total individuals	6	4	0.19	0.68

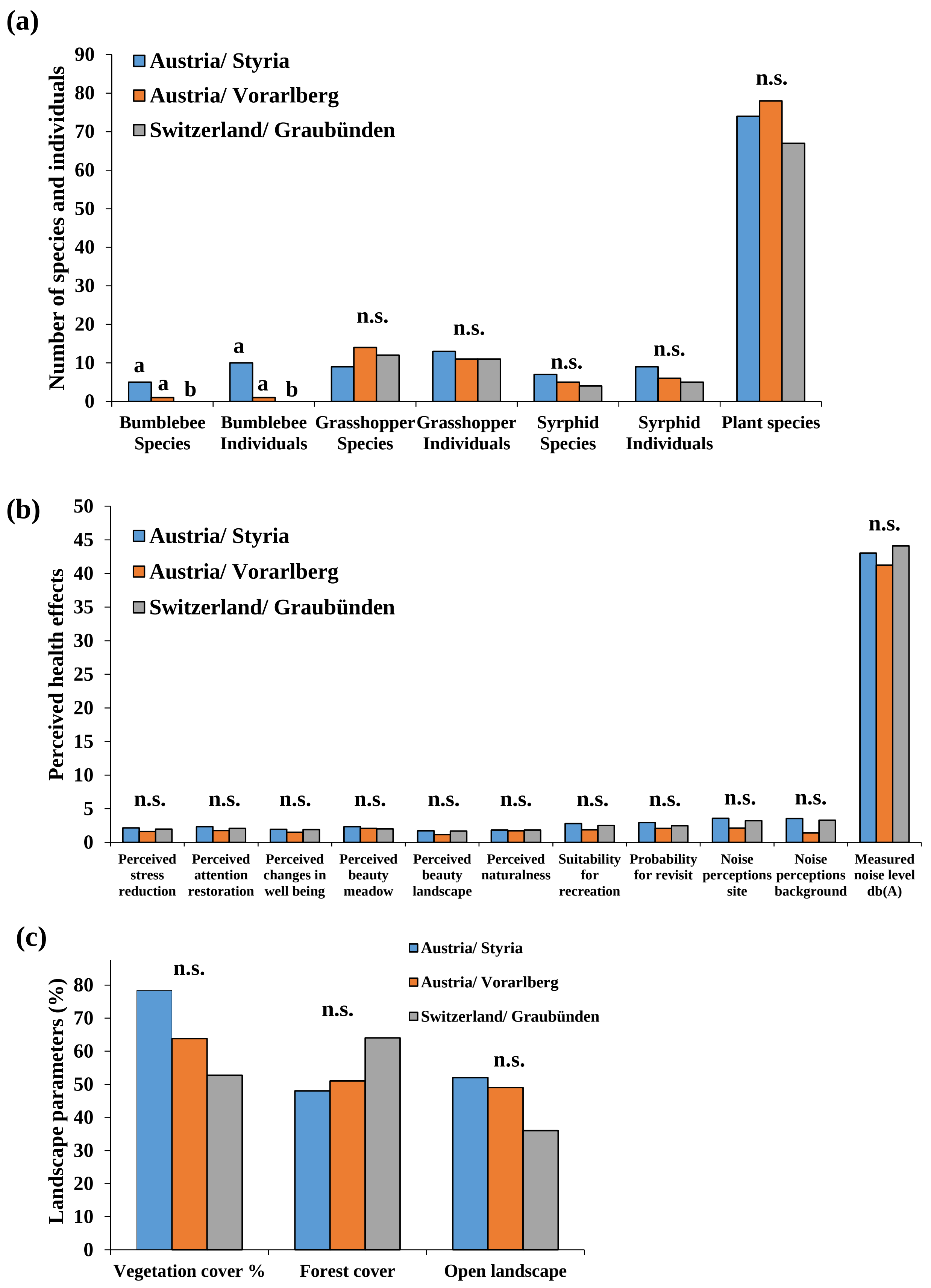

Supplementary Figure S1: (a) Biodiversity attributes, (b) perceived health effects and (c) landscape composition in three regions of Austria and Switzerland. Different letters show significant relationship ($p < 0.05$): n.s. no significant difference.
